# Phosphoglycerol-type wall and lipoteichoic acids are enantiomeric polymers differentiated by the stereospecific glycerophosphodiesterase GlpQ

**DOI:** 10.1074/jbc.RA120.012566

**Published:** 2020-02-11

**Authors:** Axel Walter, Sandra Unsleber, Jeanine Rismondo, Ana Maria Jorge, Andreas Peschel, Angelika Gründling, Christoph Mayer

**Affiliations:** ‡ Microbiology/Glycobiology, Interfaculty Institute of Microbiology and Infection Medicine Tübingen, University of Tübingen, 72076 Tübingen, Germany; § Section of Molecular Microbiology and Medical Research Council Centre for Molecular Bacteriology and Infection, Imperial College London, London SW7 2AZ, United Kingdom; ¶ Infection Biology, Interfaculty Institute of Microbiology and Infection Medicine Tübingen, University of Tübingen, 72076 Tübingen, Germany

**Keywords:** teichoic acid, cell wall, glycobiology, microbiology, Bacillus, Gram-positive bacteria, glycerolphosphate, lipoteichoic acid (LTA), stereochemistry, teichoicase, wall teichoic acid (WTA)

## Abstract

The cell envelope of Gram-positive bacteria generally comprises two types of polyanionic polymers linked to either peptidoglycan (wall teichoic acids; WTA) or to membrane glycolipids (lipoteichoic acids; LTA). In some bacteria, including *Bacillus subtilis* strain 168, both WTA and LTA are glycerolphosphate polymers yet are synthesized through different pathways and have distinct but incompletely understood morphogenetic functions during cell elongation and division. We show here that the exolytic *sn*-glycerol-3-phosphodiesterase GlpQ can discriminate between *B. subtilis* WTA and LTA. GlpQ completely degraded unsubstituted WTA, which lacks substituents at the glycerol residues, by sequentially removing glycerolphosphates from the free end of the polymer up to the peptidoglycan linker. In contrast, GlpQ could not degrade unsubstituted LTA unless it was partially precleaved, allowing access of GlpQ to the other end of the polymer, which, in the intact molecule, is protected by a connection to the lipid anchor. Differences in stereochemistry between WTA and LTA have been suggested previously on the basis of differences in their biosynthetic precursors and chemical degradation products. The differential cleavage of WTA and LTA by GlpQ reported here represents the first direct evidence that they are enantiomeric polymers: WTA is made of *sn*-glycerol-3-phosphate, and LTA is made of *sn*-glycerol-1-phosphate. Their distinct stereochemistries reflect the dissimilar physiological and immunogenic properties of WTA and LTA. It also enables differential degradation of the two polymers within the same envelope compartment *in vivo*, particularly under phosphate-limiting conditions, when *B. subtilis* specifically degrades WTA and replaces it with phosphate-free teichuronic acids.

## Introduction

The cell membrane of bacteria is covered by a complex multilayered cell envelope that protects the susceptible protoplast from lysis and from detrimental effects of the environment (1). Based on the composition of the cell envelope, bacteria are classified into two major groups: Gram-negative and Gram-positive. Gram-negative bacteria are encased in a thin peptidoglycan (PGN)^5^ layer that is covered by an external outer membrane, carrying negatively charged lipopolysaccharide in the outer leaflet. In contrast, Gram-positive bacteria lack an outer membrane but possess a thick PGN layer that is interweaved by polyanionic glycopolymers, teichoic acids, which were discovered by Baddiley and co-workers 60 years ago (2–4). Teichoic acids can be very variable in composition and structure, although they mostly feature glycerolphosphate, ribitolphosphate, or sugar phosphate repeating units connected through phosphodiester bonds (5–8). These phosphodiester polymers are either covalently bound to the PGN and called wall teichoic acids (WTA) or linked to glycolipids in the cell membrane and named lipoteichoic acids (LTA) (4, 9, 10). WTA are characteristic constituents of the Gram-positive cell walls (PGN–WTA complex), comprising chains of 30–50 polyolphosphate repeats anchored via a linker disaccharide (*N*-acetylmannosamine-β-1,4-GlcNAc (ManNAc-β-1,4-GlcNAc)) to about every ninth *N*-acetylmuramic acid residue of the PGN (9). They make up about half of the cell wall dry weight (11, 12) and are responsible for the generally high phosphate content of Gram-positive cell walls (4, 13). It has been shown that WTA can serve as phosphate storage, allowing *Bacillus subtilis* to continue growth under phosphate-depleted conditions (13–15). To cope with this stress, teichoic acids are exchanged with phosphate-free teichuronic acids in an adaptation process known as the “teichoic acid–to–teichuronic acid switch.” LTA are more widespread in bacteria than WTAs, and their composition is less dependent on growth conditions (16). Commonly, LTA contain polyol-phosphate chains (type I LTA) that are anchored to the cytoplasmic membrane via glycolipids; in the case of *B. subtilis,* a gentibiosyl disaccharide (glucose-β-1,6-glucose) β-glycosidically bound to diacylglycerol (10). WTA and LTA differ in chemical composition, cellular compartmentation and route of biosynthesis, yet their distinguishable physiological roles are insufficiently understood (4, 17–19). Although inactivation of both LTA and WTA is lethal in *B. subtilis*, indicating partially redundant functions, comparison of the individual mutants suggests differential roles during cell elongation (WTA) and division (LTA) (18). Further proposed functions of teichoic acids include control of cell wall–targeting enzymes during envelope homeostasis and divalent cation binding (3, 20), interaction with host and bacteriophage receptors (18, 21), as well as pathogenicity (22–24). Recently, LTA have been suggested to functionally resemble the osmoregulated periplasmic glycans of Gram-negative bacteria (10, 25, 26).

**Figure 1.**
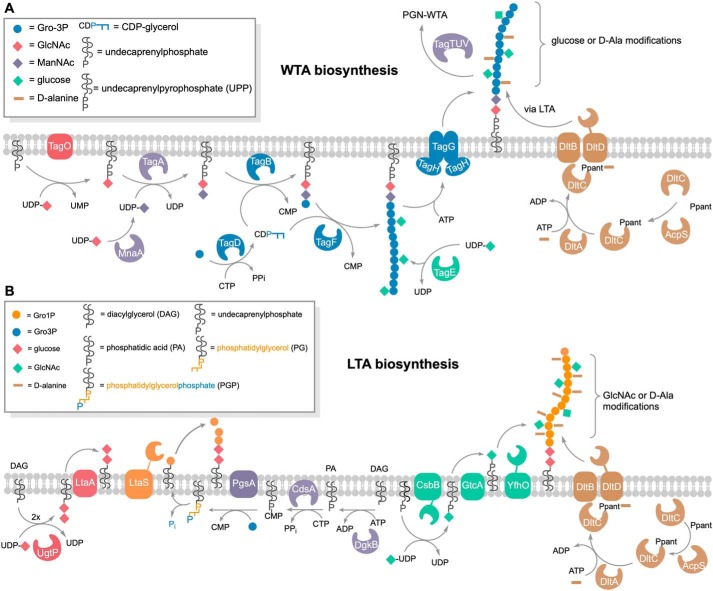
**Comparison of the biosynthetic pathways of WTA and LTA.** *A*, overview of WTA biosynthesis. TagO initiates WTA biosynthesis by transferring GlcNAc (*red diamonds*) from UDP-GlcNAc onto the lipid carrier undecaprenyl phosphate while releasing UMP. MnaA converts UDP-GlcNAc to UDP-ManNAc, which, in turn, is used to transfer ManNAc (*purple diamonds*) to the TagO product, forming the WTA linker disaccharide bound to undecaprenyl pyrophosphate (*UPP*). The TagB protein catalyzes a priming step of WTA synthesis in *B. subtilis,* completing the linkage unit (GlcNAc-ManNAc-Gro3P); a single Gro3P (*blue circles*) is added from CDP-glycerol (generated by TagD from Gro3P and CTP) to the membrane-anchored linker disaccharide while releasing CMP. TagF further elongates the WTA chain polymer by repeatedly transferring Gro3P from CDP-glycerol. The Gro3P units of the WTA polymer are partially glycosylated in the cytoplasm. The enzyme TagE utilizes UDP-glucose to attach glucose (*green diamonds*) onto the C2 hydroxyl group of Gro3P of the chain polymer. The degree of glycosylation strongly depends on growth conditions and growth phase. The WTA polymer is translocated across the cell membrane via the ABC transporter TagGH. Finally, the membrane-anchored TagTUV ligases transfer WTA polymers to PGN and the DltABCD system attaches d-alanyl esters to nonglycosylated parts of WTA in an LTA-dependent process (34). DltA transfers d-alanine in an ATP-dependent two-step reaction to DltC, which has been modified with 4′-phosphopantetheine (*Ppant*) at Ser-35 by acyl carrier protein synthase (*AcpS*) (56). DltB interacts with DltC-Ppant and together with DltD transfers the d-alanyl onto the C2 hydroxyl group of Gro3P of teichoic acid chains (10, 57). *B*, overview of LTA biosynthesis. The LTA precursor PGP is generated by a series of reactions within the cytoplasm. Synthesis starts by phosphorylation of DAG, yielding DAG phosphate (phosphatidic acid (*PA*)). The enzyme CdsA then transfers a CMP moiety from CTP onto phosphatidic acid, yielding DAG-CDP, while releasing pyrophosphate (*PP_i_*). The CMP moiety of the latter is exchanged with Gro3P by PgsA, forming PGP. Notably, PG is formed by releasing the *sn*-3-phosphoryl group from PGP, retaining a Gro1P entity. It is unclear whether this reaction is catalyzed by a dedicated but uncharacterized phosphatase or by the LTA synthase (*LtaS*) to energize polymerization of Gro1P entities onto the DAG-anchored linker disaccharide in the outer leaflet of the plasma membrane. The linker disaccharide (*red diamonds*) is synthesized by UgtP by addition of two glucose from UDP-glucose onto DAG, which is then flipped across the membrane by LtaA (58). LTA polymers may be modified by d-alanylation (*brown*) and glycosylation (*green*). As described above, alanylation is catalyzed by the Dlt alanylation system; DltA transfers d-alanine to DltC-Ppant, and subsequently DltB together with DltD transfers d-alanyl onto C2 of Gro1P (10, 57). Glycosylation of LTA is catalyzed by the glycosyltransferase CsbB, which adds GlcNAc onto undecaprenyl phosphate (*C_55_-P*) using UDP-GlcNAc. As this modification occurs outside of the cell in *B. subtilis*, C_55_-P–GlcNAc is first flipped across the membrane by the flippase GtcA, and then the glycosyltransferase YfhO modifies C2 of Gro1P with GlcNAc (38, 40).

**Figure 2.**
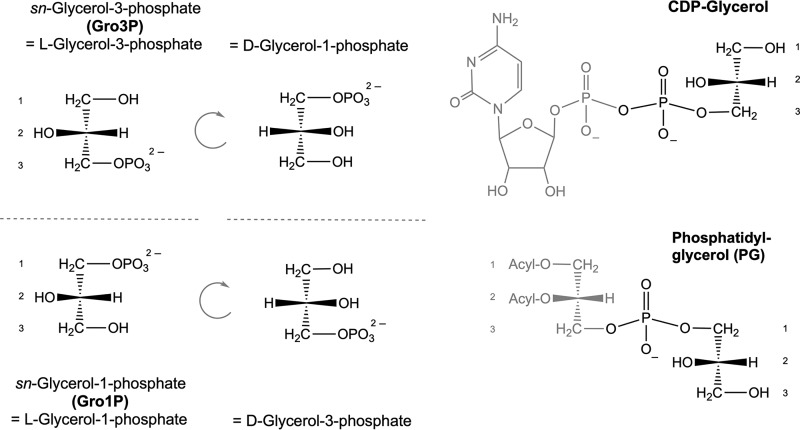
**Stereochemistry of glycerolphosphates and teichoic acid precursors.** The precursors of WTA and LTA synthesis carry enantiomeric glycerolphosphates: *sn*-glycerol-3-phosphoryl (CDP-glycerol) and *sn*-glycerol-1-phosphoryl (PG), respectively. Glycerolphosphate enantiomers are defined by convention according to stereochemical numbering (*sn* nomenclature) as *sn*-glycerol-3-phosphate (Gro3P = l-glycerol-3-phosphate = d-glycerol-1-phosphate) and *sn*-glycerol-1-phosphate (Gro1P = l-glycerol-1-phosphate = d-glycerol-3-phosphate).

In some Gram-positive bacteria, including *B. subtilis* 168, *Staphylococcus epidermidis*, and *Staphylococcus lugdunenis*, both LTA and WTA are glycerophosphate polymers (17, 18, 27, 28). Nevertheless, they are synthesized through distinct routes (9, 10, 29). WTA are synthesized from CDP-glycerol in the cytoplasm, and the polymers are then flipped outward (Fig. 1*A*). In contrast, LTA are synthesized from the precursor phosphatidylglycerol (PG), generated via diacylglycerol (DAG) CDP and PG phosphate (PGP). The PG precursor is subsequently translocated across the cell membrane and then polymerized on the outside of the cell (Fig. 1*B*). Intriguingly, the glycerophosphate in the precursors of WTA and LTA has different stereochemistry (17). The prochirality of glycerol leads to two 3-phosphate products; by convention, l-glycerol is the configuration that determines the stereochemical numbering (*sn* nomenclature) of glycerolphosphates (Fig. 2). CDP-glycerol has a *sn-*3 configuration, whereas the free glycerolphosphate of PG has a *sn-*1 configuration. The use of different precursors and the compartmentalization of their synthesis allow differential regulation of production of WTA and LTA, which is important for their specific roles in cell envelope integrity and morphogenesis (18). However, how WTA and LTA execute these distinct functions in the same cell envelope compartment is still unclear.

Modification of polyols by alanylation and glycosylation is an important means to alter the physiological properties of WTA and LTA and also affect recognition by the innate immune system (6, 30, 31). d-alanylation adds a positive charge (free amino groups) to the polyolphosphate polymers, conferring the anionic character and, as a consequence, the binding properties (10, 32). The multienzyme complex DltABCD is responsible for adding d-Ala modifications onto LTA on the outer leaflet of the cell membrane and indirectly also onto WTA (Fig. 1) (32–35). LTA and WTA can also be α- or β-glycosylated, modifications that strongly increase the stability of these polymers against alkaline hydrolysis (9, 10). In *B. subtilis*, the enzyme TagE transfers α-glucosyl residues from UDP-glucose onto preformed WTA within the cytoplasm and represents the only WTA glycosylating enzyme in this bacterium (Fig. 1*A*) (36, 37). Although WTA glycosylation usually occurs prior to translocation of the polymer across the cell membrane, it was recently proposed to also occur after translocation in *Listeria monocytogenes* (37, 38). Like alanylation, glycosylation of LTA generally occurs, along with synthesis, outside of the cell, and membrane-associated, three-component LTA glycosylation systems have recently been characterized in *B. subtilis* and *Staphylococcus aureus (*CsbB/GtcA/YfhO) as well as in *L. monocytogenes* (GtlA/GtlB) (Fig. 1*B*) (38–40).

Besides synthesis, turnover of WTA and LTA also needs to be differentially regulated, but so far this process has been poorly investigated. Recently, the exo-acting *sn*-glycero-3-phosphate phosphodiesterase GlpQ, along with an endo-acting phosphodiesterase, PhoD, has been implicated in degradation of WTA during phosphate starvation (41). However, apart from WTA degradation during adaptation to phosphate starvation, turnover of WTA likely occurs along with turnover of PGN of the cell wall in *B. subtilis* and other Gram-positive bacteria (42–44). Because strains of *B. subtilis* lacking both WTA and LTA are not viable, simultaneous degradation of both polymers would be detrimental (18). We thus wondered how differential degradation of WTA and LTA by hydrolases (“teichoicases”) is regulated. Previous studies with the glycerophosphodiesterase GlpQ of *B. subtilis* as well as orthologous enzymes from *Escherichia coli* and *S. aureus* (amino acid sequence identities of 29% and 54%, respectively) have revealed strict stereospecificity for glycerophosphodiesters harboring *sn*-glycerol-3-phosphoryl groups, *e.g.* produced by phospholipases from membrane phospholipids (41, 45–47). Accordingly, phosphatidylglycerol or lysophosphatidylglycerol, which harbor only free *sn*-glycerol-1-phosphoryl ends, are not hydrolyzed by GlpQ, and bis(*p*-nitrophenyl) phosphate, a chromogenic substrate for other phosphodiesterases, is also not cleaved by GlpQ (45, 46). Intriguingly, GlpQ is also unable to hydrolyze LTA of *S. aureus*, but this could be due to the presence of modifications on the phosphoglycerol backbone (47). In contrast, the enzyme shows broad substrate specificity with respect to the alcohol moiety and can hydrolyze a variety of different phospholipid headgroups, such as glycerophosphocholine, glycerophospho-ethanolamine, glycerophosphoglycerol, and bis(glycerophospho)-glycerol (41, 45, 47).

So far, differential cleavage of WTA and LTA polymers by GlpQ has not been examined in detail. In this work, we show that the stereospecific *sn*-glycerol-3P phosphodiesterase GlpQ acts as an exolytic hydrolase that sequentially cleaves off *sn*-glycerol-3-phosphate (Gro3P) entities from the exposed end of WTA but is unable to hydrolyze intact LTA. Thereby, we provide biochemical evidence that these polymers have opposite stereochemistry: WTA constitute phosphodiester-polymers made of Gro3P and LTA polymers of *sn*-glycerol-1-phosphate (Gro1P). This stereochemical difference likely determines many of the polymers' distinct properties, such as interactions with hydrolases and binding of proteins throughout the cell cycle, bacterial growth, and differentiation.

## Results and discussion

### GlpQ is a stereospecific sn-glycerol-3-phosphoryl phosphodiesterase

GlpQ of *B. subtilis* and orthologs from other bacteria have been shown previously to specifically release Gro3P from *sn*-glycero-3-phosphocholine (GPC), glycerophosphoethanol amine, glycerophosphoglycerol, and bis(glycero-phospho)glycerol. For the latter two substrates, *K_m_* and *k*_cat_ values of 1.0 mm and 1275 min^−1^ and, respectively, 1.4 mm and 1517 min^−1^ were determined for *B. subtilis* GlpQ (41, 45, 47). We confirmed the stereospecificity of recombinant *B. subtilis* GlpQ for *sn*-glycero-3-phosphoryl substrates and determined the enzyme's stability and catalytic optima using GPC as substrate (Fig. S1). Our analysis revealed that GlpQ is rather temperature-sensitive. It readily loses stability at temperatures above 30 °C; more than 50% of its activity was lost within 30 min at 37 °C. At the same time, however, enzymatic turnover steadily increased with temperature up to an optimum at 55 °C with about half-maximum activity at 30 °C. Furthermore, the enzyme was shown to be stable over a remarkably wide pH range, between 2 and 10, but had a very narrow optimum at pH 8.0 (Fig. S1*B*). We thus conducted all experiments with the enzyme GlpQ in this study at 30 °C and pH 8.0.

**Figure 3.**
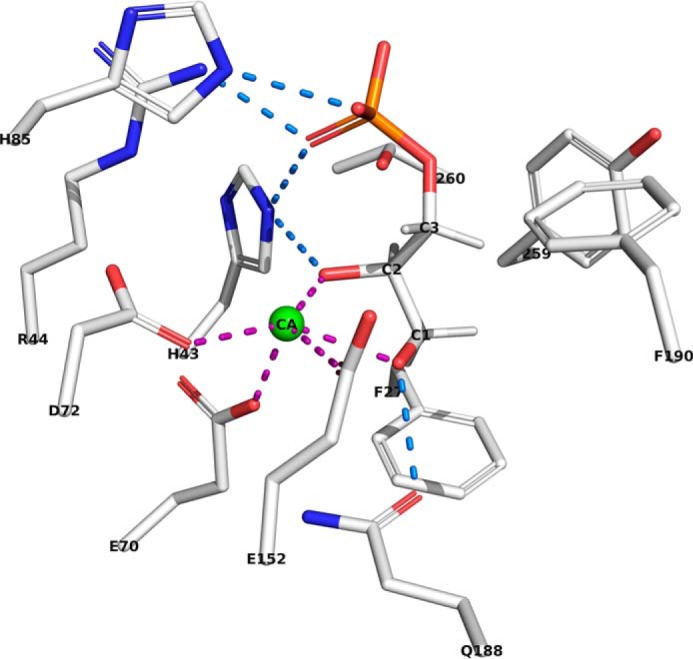
**The cocrystal structure of GlpQ in complex with Gro3P rationalizes the strict stereospecificity of GlpQ for *sn*-glycero-3-phosphoryl groups.** In the cocrystal structure of Myers *et al.* (PDB code 5T9B (41)), the active site of GlpQ features a hydrophilic side with His-85, His-43, Arg-44, Asp-72, Gln-188, Glu-152. and Glu-70 (*left* of Gro3P; carbon chains, *gray*; oxygens, *red*; phosphor, *orange*; nitrogens, *blue*). The other side of the binding cleft (*right* of Gro3P) consists of hydrophobic amino acids like phenylalanine, tyrosine, and leucine (Phe-190, Tyr-259, and Phe-279). The Ca^2+^ ion adopts a pentagonal bipyramidal coordination and is coordinated by Glu-70, Glu-152, and Asp-72 as well as by the two hydroxyl groups of Gro3P. The phosphate of the Gro3P substrate makes hydrogen bond interactions with Arg-44, His-43, and His-85. The C2 hydroxyl group of Gro3P interacts with Ca^2+^ and His-43. Further, the C3 hydroxyl group of the substrate binds to Ca^2+^ and Gln-188.

Although the detailed mechanism of phosphodiester cleavage by GlpQ is currently unknown, Ca^2+^ ions were recognized as crucial for catalytic activity (but they can be substituted with Cd^2+^ and partially with Mn^2+^ and Cu^2+^) (45, 48). Accordingly, the catalytic reaction was inhibited with EDTA. Nevertheless, addition of Ca^2+^ ions was not required when using the recombinant GlpQ that was purified from the cytosolic extracts of *E. coli*. The recently solved crystal structure of *B. subtilis* GlpQ with Gro3P bound to the active site (PDB codes 5T9B and 5T9C) confirmed the importance of a Ca^2+^ ion for catalysis as well as for the stereospecific coordination of the substrate (41, 48). The active site of GlpQ includes a residue (His-85) that is located on a small additional, so-called glycerophosphodiester phosphodiesterase domain that is inserted between the β-strand and α-helix of the second β/α motif of a classical triose phosphate isomerase barrel structure (41, 49). As shown in Fig. 3, the substrate binding cleft can be divided into a hydrophilic side, including the active-site Ca^2+^ ion, and a hydrophobic side consisting of hydrophobic amino acids, including phenylalanine and tyrosine (Phe-190, Tyr-259, and Phe-279). The active site Ca^2+^ ion adopts a pentagonal bipyramidal coordination; it is held in place by glutamic and aspartic acid residues (Glu-70, Glu-152, and Asp-72) and is also coordinated by the two hydroxyl groups of Gro3P (Fig. 3). The phosphate as well as the hydroxyl groups at C2 and C3 of Gro3P are drawn toward the Ca^2+^ ion in the active site and moved away from the hydrophobic side of the binding cleft. Coordination of the Ca^2+^ ion by amino acids with charged side chains and the hydroxyl and phosphate groups of the substrate as well as the orientation of the hydrophobic C-H groups of the substrate toward the hydrophobic side of the binding cleft restrict productive binding to the unsubstituted *sn*-glycero-3-phosphoryl stereoisomer, only allowing hydrolysis of *sn*-glycerol-3-phosphoryl groups. Instead, the C2 hydroxyl group of *sn*-glycerol-1-phosphoryl would face toward the hydrophobic side, precluding productive binding. The hydrophilic side of the binding cleft also coordinates the phosphate group of the substrate involving the basic side chains of His-43, Arg-44, and His-85 (Fig. 3). His-43 and His-85 presumably function as general acid and base residues in the mechanism of phosphodiester hydrolysis (48). The proposed catalytic mechanism of GlpQ involves anchimeric assistance of the C2 hydroxyl group, requiring this group to be unmodified, *i.e.* not glycosylated or alanylated at the C2 hydroxyl group of GroP (41).

### GlpQ sequentially cleaves unmodified WTA by an exolytic mechanism

**Figure 4.**
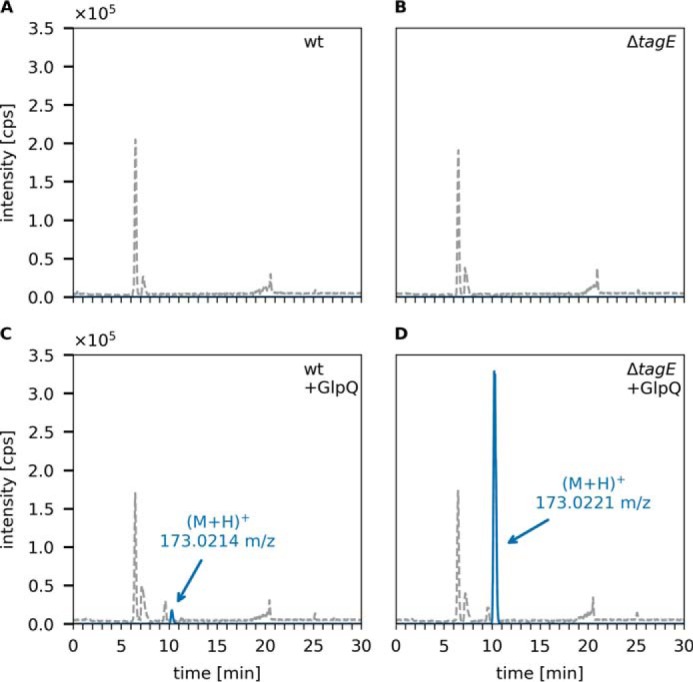
**GlpQ predominantly releases Gro3P from the cell walls of Δ*tagE B. subtilis* 168.** The purified cell wall of *B. subtilis* (containing peptidoglycan and covalently bound WTA (PGN-WTA complex)) was incubated with GlpQ, and the formation of reaction products was analyzed by LC-MS. Shown are the BPC for mass range (M+H)^+^ = 120–800 (*gray dashed lines*) and the EICs of glycerolphosphate (M+H)^+^ *m*/*z* = 173.022 ± 0.02 (*blue solid lines*). *A* and *B*, analysis of the WT containing partially glycosylated WTA and Δ*tagE* containing nonglycosylated WTA cell walls in the absence of GlpQ (control). *C* and *D*, analysis of processing of WT and Δ*tagE* cell walls after 30-min incubation with GlpQ. The peak areas (AUC) of released GroP were 2.7 × 10^5^ and 5.9 × 10^6^, respectively. No glycosylated or alanylated GroP products were detected.

The glycerophosphodiesterase GlpQ of *B. subtilis* has recently been identified as a teichoicase that preferentially digests polyGroP-type WTA that lack modifications on the glycerol subunits (41). However, in this study, the product of digestion of polymeric teichoic acids by GlpQ was not monitored. Hence, neither the strict specificity for unmodified WTA nor the exolytic mechanism have been shown unequivocally. We thus aimed to directly monitor product release by GlpQ from cell wall (PGN–WTA complex) preparations using HPLC-MS. We first applied cell wall preparations containing modified (glycosylated) WTA extracted from *B. subtilis* 168 WT cells and cell wall preparations containing unmodified (nonglycosylated) WTA extracted from Δ*tagE::erm* cells that lack the WTA α-glucosyl transferase TagE (*cf.* Fig. 1*A*). These samples were digested with GlpQ, and product formation was followed by HPLC-MS. In both cell wall preparations, GroP was detected in the presence but not in the absence of GlpQ (Fig. 4). However, GlpQ released large amounts of GroP from Δ*tagE::erm* cell wall samples and very little GroP from WT cell wall samples (Fig. 4). The amounts of GroP, determined by calculating the area under the curve (AUC), were about 22 times higher when applying cell walls containing nonglycosylated WTA (AUC = 5.9 × 10^6^) compared with cell walls containing glycosylated WTA prepared from WT cells (AUC = 2.7 × 10^5^), which is in agreement with the proposed chain length of the WTA polymers of 30–50 polyolphosphate repeats. The identity of the GroP reaction product was confirmed by MS via the exact mass and typical adduct pattern and isotope profiles for GroP (Fig. S2*A*). It should be noted that it is not possible with the HPLC-MS method to discriminate between the two stereoisomers of GroP (*cf.* Fig. 2); however, given the strict stereospecificity of GlpQ, the product of WTA cleavage has to be *sn*-glycerol-3-phosphate. The little amount of GroP detected in WT cell wall samples is presumably the result of the activity of GlpQ on nonglycosylated GroP at the free ends of the substrate. As GlpQ encounters a glycosylated (or alanylated) GroP in the chain polymer, the hydrolysis reaction and, consequently, GroP release stop. This hypothesis is corroborated by the finding that neither glycosylated GroP-Glc or alanylated GroP-Ala nor larger polymeric products but only unmodified GroP could be detected by HPLC-MS. Thus, GlpQ can be classified as a teichoicase that specifically hydrolyzes unmodified *sn*-glycero-3-phosphoryl–WTA.

In further support of this model, d-Ala substitutions were removed from the teichoic acid samples by pretreatment as well as by applying the GlpQ reaction at pH 8. It has been reported earlier that alanyl esters are rather labile at pH levels of 7 or higher, with a half-time of hydrolysis at pH 8 and 37 °C of 3.9 h (32, 50). Accordingly, no difference in release of GroP was observed between nontreated and pH 8–pretreated WTA samples (data not shown). Furthermore, in a time course experiment, we observed that the majority of the product in both glycosylated (WT cell–extracted) and nonglycosylated (Δ*tagE* cell–extracted) samples was released by GlpQ already after a few seconds (Fig. S3). Moreover, the amount of GroP released from nonglycosylated substrate did not increase over time (over 2 h of incubation) and remained 22-fold higher than the product released from the WT substrate. These data indicate that GlpQ has only exo- but no endolytic activity and stops when glycosylated (or alanylated) GroP appears at the free end of the polymer, protecting the rest of the chain from further digestion.

**Figure 5.**
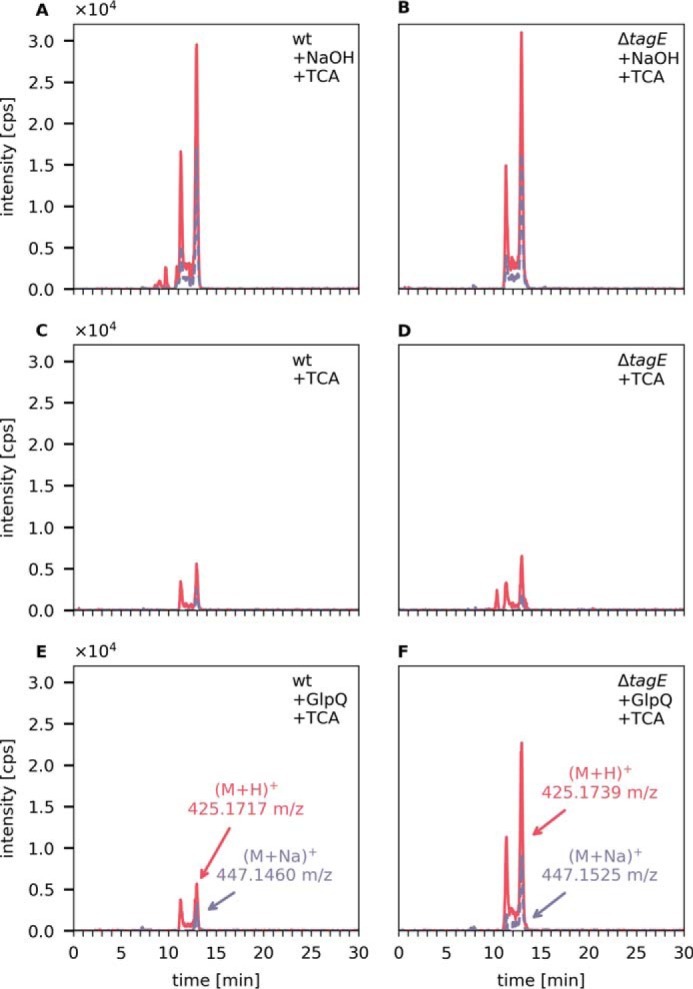
**GlpQ completely digests nonglycosylated WTA up to the linker disaccharide ManNAc-GlcNAc.** Purified cell walls (PGN–WTA complex, 0.1 mg each) of *B. subtilis* 168 WT and Δ*tagE* were repeatedly incubated with GlpQ (seven times for 10 min at 30 °C) and then treated with 5% TCA for 2 h at 60 °C to release the linker disaccharide. The release of ManNAc-GlcNAc was analyzed by LC-MS. As a control, the cell wall was treated with 0.5 m NaOH for 2 h at 60 °C to release all GroP from the WTA chain polymers, followed by TCA treatment to release the linker disaccharide. Shown are the EICs of ManNAc-GlcNAc (M+H)^+^ *m*/*z* = 425.177 ± 0.02 (*red solid lines*) and (M+Na)^+^ *m*/*z* = 447.159 ± 0.02 (*purple dashed lines*). *A* and *B*, complete release of the linker disaccharide after NaOH and TCA treatment from WT cell walls (AUC = 15.1 × 10^5^) and from Δ*tagE* cell walls containing nonglycosylated WTA (AUC = 14.8 × 10^5^). *C* and *D*, linker disaccharide released by TCA treatment alone from WT cell walls (AUC = 2.38 × 10^5^) and Δ*tagE* cell walls (AUC = 3.2 × 10^5^). *E* and *F*, linker disaccharide released by TCA treatment after predigestion with GlpQ from WT cell walls (AUC = 2.84 × 10^5^, *i.e.* 3.6% of the total linker disaccharide) and Δ*tagE* cell wall (AUC = 1.0 × 10^6^, *i.e.* 59% of the totally present linker disaccharide).

Complete digestion of WTA by GlpQ should remove all GroP residues up to the linker disaccharide ManNAc-GlcNAc. To show that this is indeed the case, cell wall preparations (PGN–WTA complex) were thoroughly digested by GlpQ. As the enzyme is rather unstable, GlpQ was added repeatedly; after each round of enzymatic digestion for 10 min at 30 °C, the supernatant was checked for GroP release by HPLC-MS and fresh GlpQ was enzyme added until only very minor additional amounts of GroP were detected. These exhaustively digested cell wall samples were then treated with 5% TCA for 2 h at 60 °C to enable cleavage of the glycosidic phosphodiester bond connecting the WTA linker with the PGN. The release of the linker disaccharide was analyzed by HPLC-MS after neutralization of the sample. The identity of the linker disaccharide was confirmed by a mass spectrum that revealed the exact mass and presence of typical fragmentations (loss of water), sodium and potassium ion adducts, and a ^13^C isotope pattern (Fig. S2*B*). As control, complete chemical digestion of the PGN–WTA complex was performed by treatment with 0.5 m NaOH for 2 h at 60 °C to completely remove the GroP chain polymer. Subsequently, the linker disaccharide was released from the latter samples by TCA treatment and analyzed by HPLC-MS. The linker disaccharide was obtained from both WT and nonglycosylated PGN–WTA complexes by chemical digestion in equal amounts, as shown in Fig. 5, *A* and *B*. The amount of linker disaccharide released by chemical digestion was set as to 100% of linker disaccharide in the substrate. As a further control, the PGN–WTA complex was treated with TCA alone to determine the amounts of linker disaccharide TCA can release in absence of NaOH pretreatment. Very small amounts of linker disaccharide (approximately 3.6% of the total) were released from both PGN–WTA variants under these conditions (Fig. 5, *C* and *D*). The difference, however, became significant when the substrate was predigested with GlpQ. Although GlpQ treatment released no more linker than TCA treatment alone from WT PGN–WTA, GlpQ was able to digest about 60% of WTA up to the linker in the cell wall sample derived from *tagE* mutant cells (Fig. 5, *E* and *F*).

### GlpQ cleaves unmodified LTA only after predigestion

Because GlpQ specifically cleaves nonglycosylated WTA, we next assessed whether nonglycosylated LTA can also act as a substrate of the enzyme. Recently, the glycosyltransferase CsbB has been shown to be required for glycosylation of LTA in *B. subtilis* (38). Hence, LTA was purified from *B. subtilis* WT and Δ*csbB::kan* cells according to established protocols (51, 52). Because LTA has been reported to be extensively modified by d-alanyl esters, we set out to also remove these modifications prior to GlpQ treatment. Although incubation of LTA at pH 8.5 for 24 h at room temperature leads to almost complete removal of d-alanyl esters, it may also induce limited degradation of LTA according to data reported previously (22). Hence, to absolutely avoid any degradation of LTA, we decided to apply slightly milder conditions and preincubated the LTA preparations in borate buffer at pH 8 for 24 h. The removal of alanine modifications was monitored by NMR (Fig. S5). The ^1^H NMR spectra of LTA showed characteristic resonances corresponding to d-alanyl ester modifications; the signal at δ = 5.35, 4.20, 1.64, and 4.2 ppm could be assigned to resonances of Gro-2-CH (d-Ala), d-Ala–βH, and d-Ala–αH, respectively. These resonances decreased significantly and shifted, indicating release of d-Ala from the GroP polymer. According to the NMR results, about 70% of the d-alanyl esters were removed by treatment of *B. subtilis* 168 LTA in borate buffer at pH 8 for 24 h at room temperature.

**Figure 6.**
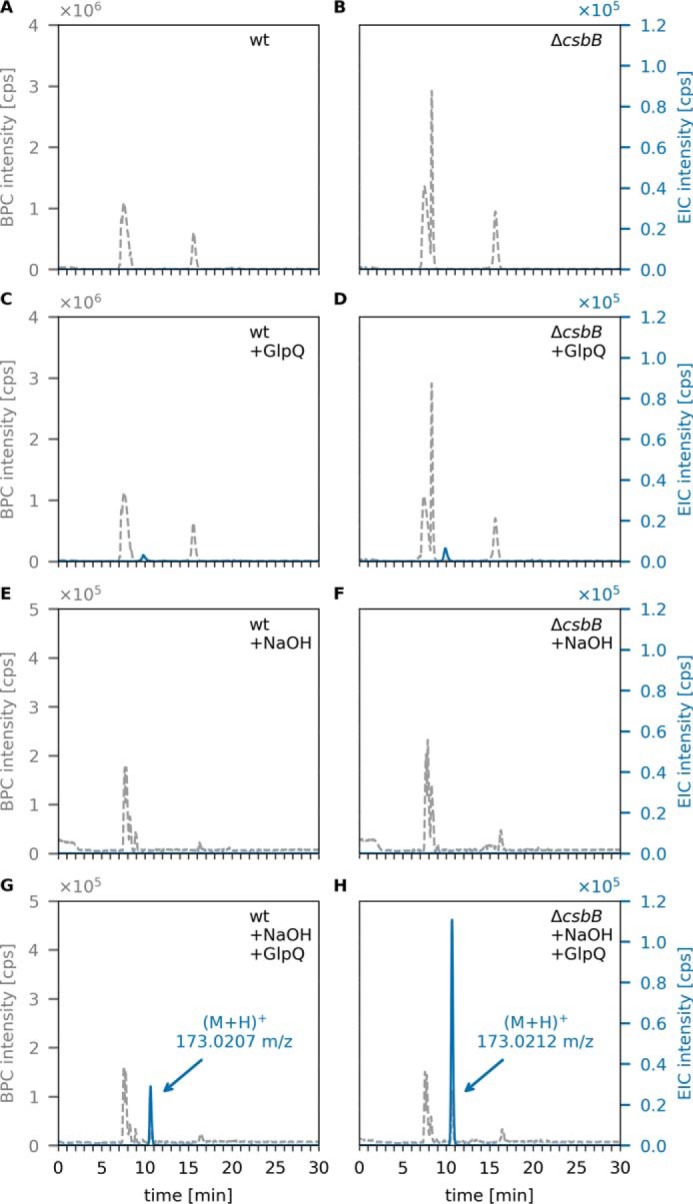
**GlpQ releases *sn*-glycerol-3P from NaOH-pretreated LTA of *B. subtilis* WT and Δ*csbB* mutant cells.** Purified *B. subtilis* LTA was incubated for 24 h at room temperature and pH 8, followed by incubation with GlpQ. The formation of reaction products was analyzed by LC-MS. Shown are the BPC mass range (M+H)^+^ = 120–800 (*gray dashed lines*) and EICs of glycerolphosphate (M+H)^+^ *m*/*z* = 173.022 ± 0.02 (*blue solid lines*). *A* and *C*, WT LTA (partially glycosylated LTA) incubated without GlpQ (control) and with GlpQ. The peak area of released GroP was AUC = 1 × 10^5^. *B* and *D*, nonglycosylated Δ*csbB* LTA incubated without GlpQ (control) and with GlpQ. The peak area of released GroP was AUC = 1.8 × 10^5^. *E* and *G*, WT LTA pretreated with NaOH incubated without GlpQ (+NaOH) and with GlpQ. The peak area of released GroP was AUC = 4.6 × 10^5^. *F* and *H*, nonglycosylated Δ*csbB* LTA pretreated with NaOH incubated without GlpQ (+NaOH) and with GlpQ. The peak area of released GroP was AUC = 1.69 × 10^6^.

**Figure 7.**
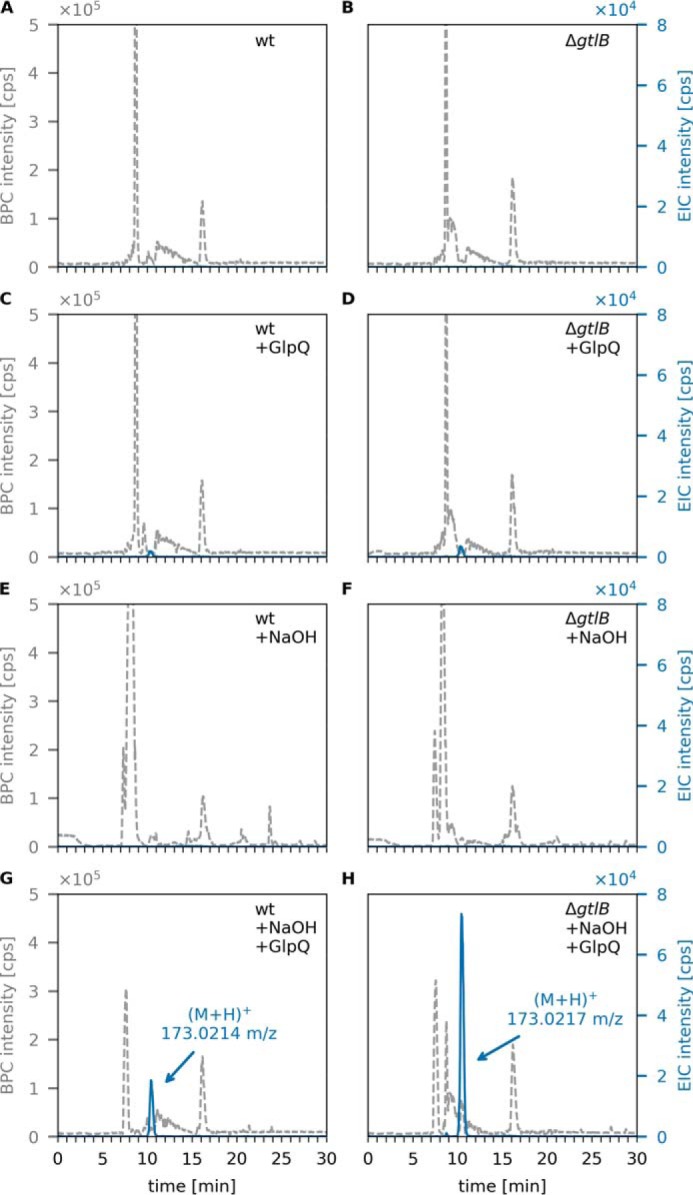
**GlpQ releases *sn*-glycerol-3P from NaOH-pretreated LTA of *L. monocytogenes* WT and Δ*gtlB* mutant cells.** Purified *L. monocytogenes* LTA was incubated with GlpQ, and the formation of reaction products was analyzed by LC-MS. Shown are the BPC mass range (M+H)^+^ = 120–800 (*gray dashed lines*) and EICs of glycerolphosphate (M+H)^+^ *m*/*z* = 173.022 ± 0.02 (*blue solid lines*). *A* and *C*, WT LTA (partially glycosylated LTA) incubated without GlpQ (control) and with GlpQ. The peak area of released GroP was AUC = 6 × 10^4^. *B* and *D*, nonglycosylated Δ*gtlB* LTA incubated without GlpQ (control) and with GlpQ. The peak area of released GroP was AUC = 1.2 × 10^5^. *E* and *G*, WT LTA pretreated with NaOH incubated without GlpQ (+NaOH) and with GlpQ. The peak area of released GroP was AUC = 4.5 × 10^5^. *F* and *H*, nonglycosylated Δ*gtlB* LTA pretreated with NaOH incubated without GlpQ (+NaOH) and with GlpQ. The peak area of released GroP was AUC = 1.87 × 10^6^.

**Figure 8.**
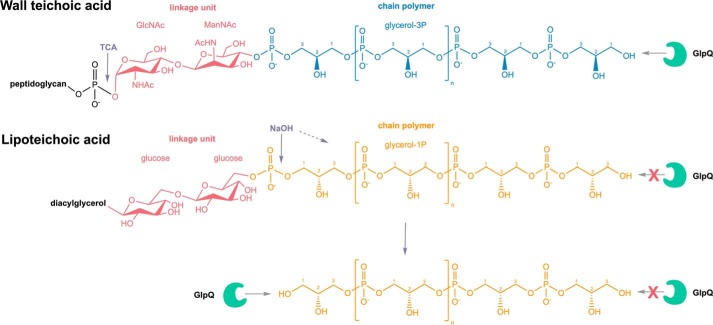
**Differential digestion of WTA and LTA by the stereospecific *sn*-glycerol-3P phosphodiesterase GlpQ.** WTA of *B. subtilis* 168 are phosphodiester polymers made of Gro3P subunits that are generally substituted at the hydroxyl group at the C2 position to a certain degree with d-alanine or alpha-glucose. Via a linkage unit (*red*) consisting of a disaccharide, ManNAc-β(1–4)-GlcNAc, and an unsubstituted Gro3P, WTA are linked to the C6 of *N*-acetylmuramic acid of the peptidoglycan via a phosphodiester bond. Trichloroacetic acid (TCA) treatment enables cleavage of the glycosidic phosphodiester bond that connects the WTA linker with the PGN. LTA of *B. subtilis* 168 is a phosphodiester polymer made of Gro1P subunits; hence, it represents an enantiomer of the WTA polymer. It can be modified at the C2 position with d-alanine or GlcNAc and linked to a diacylglycerol via a glucose-β-1,6-glucose linker disaccharide. GlpQ is able to cleave off Gro3P from the terminal ends of WTA. Conversely, GlpQ is not able to chip off Gro1P from the terminal ends of LTA. The stereospecific enzyme GlpQ is able to discriminate the orientation of the hydroxyl group on the C2. Treatment with NaOH enables precleavage of phosphodiester bonds within the LTA chain polymer, resulting in fragments that contain Gro3P terminal ends. From these ends, GlpQ is able to cleave off Gro3P moieties. The differential cleavage of WTA and LTA by GlpQ unravels the different stereochemistry of the polymers.

Only very small amounts of GroP were released by GlpQ from LTA extracted from WT or Δ*csbB* mutant cells, with AUC values of 1.0 × 10^5^ and 1.8 × 10^5^, respectively (Fig. 6). In the absence of preincubation under mildly alkaline conditions (borate buffer (pH 8), 24 h), the amount of GroP released by GlpQ did not change (Fig. S4). When LTA extracts were incubated under alkaline conditions (0.1 m NaOH, 60 °C, 30 min) in the absence of GlpQ to partially hydrolyze phosphodiester bonds within the polymer, no GroP could be detected, indicating little degradation of LTA (Fig. 6, *E* and *F*). Subsequent addition of GlpQ, however, released substantial amounts of GroP, particularly from nonglycosylated LTA preparations (Fig. 6, *G* and *H*). The amount of GroP released by GlpQ was about 3.7 times higher with nonglycosylated LTA (AUC = 1.69 × 10^6^) compared with WT glycosylated LTA (AUC = 4.6 × 10^5^). The same pattern could be observed for LTA obtained from *L. monocytogenes* WT and LTA glycosylation-deficient (Δ*gtlB*) strains. Although GlpQ released only small amounts of GroP from WT (AUC = 6 × 10^4^) and nonglycosylated (Δ*gtlB*) (AUC = 1.2 × 10^5^) LTA (Figs. 7, *C* and *D*), the amount increased significantly after NaOH pretreatment (Fig. 7, *G* and *H*) with 4.2 times more GroP released from nonglycosylated LTA (AUC = 1.87 × 10^6^) than from the WT (AUC = 4.5 × 10^5^). These results indicate that GlpQ is only able to release significant amounts of GroP from LTA when the polymer is precleaved with NaOH, which generates LTA fragments that expose *sn*-glycero-3-phosphoryl groups at the free ends (Fig. 8). The low amounts of GroP that are released by GlpQ from LTA preparations under mildly alkaline conditions may be the result of partial phosphodiester cleavage of the polymer at pH 8.0. However, phosphodiester cleavage under these conditions is unlikely, and, consistently, we were unable to detect LTA degradation by ^1^H NMR analysis. The most likely explanation is that low amounts of GroP are released by GlpQ from the free *sn*-glycero-3-phosphoryl ends of lipid II–bound WTA precursors, which are copurified with LTA on a hydrophobic interaction column during sample preparation. It could be argued that GlpQ may not be able to degrade LTA because of limited accessibility to the membrane-bound substrate and that the enzyme only works well when LTA is predigested, which removes the membrane anchor. However, LTA is water-soluble, and there is no reason to doubt that GlpQ can have access to the hydrophilic free ends of the polymer. Moreover, it was shown that membrane-anchored precursor molecules of WTA are readily cleaved by GlpQ (41). Clearly, substitutions at GroP by glycosylation or alanylation impede the action of GlpQ, leaving the question of how substituted WTA (and LTA) may be processed. Presumably, the cells have additional enzymes that can act on substituted teichoic acids. PhoD of *B. subtilis*, recently identified as an endolytic teichoicase, is able to cleave glycosylated WTA (41).

In summary, GlpQ releases GroP in significant amounts from WTA (Fig. 4) but only very small amounts from LTA preparations (Figs. 6*C* and 7*C*). These findings experimentally confirm differences in the stereochemistry of the two polyglycerolphosphate polymers. In agreement with the described stereospecificity of GlpQ, WTA consist of Gro3P- and LTA of Gro1P-repeating units. The origin of this difference can be found in the early stages of biosynthesis. WTA biosynthesis starts with Gro3P, which is transferred to CTP by TagD with simultaneous release of pyrophosphate, generating CDP-glycerol. The chain polymer is elongated in the cytoplasm with addition of Gro3P to the growing chain, and CMP is released (Fig. 1*A*) (53). In contrast, LTA biosynthesis starts with phosphatidylglycerol-CMP, onto which PgsA transfers Gro3P while releasing CMP. The 3P group of Gro3P is released, and the product PG is translocated across the membrane and polymerized. The glycerolphosphate group of PG carries a 1-phosphate group, incorporating Gro1P in the growing LTA chains (Fig. 1*B*) (10).

### Conclusions

Our work reveals the distinct stereoisomerism of the glycerophosphate polymers WTA and LTA of *B. subtilis* by differential digestion with the stereospecific phosphodiesterase GlpQ. First, we were able to show that the stereospecific *sn*-glycerol-3P phosphodiesterase GlpQ is an exolytic hydrolase that sequentially cleaves off GroP entities from unmodified WTA, which lack any modification in form of d-alanylation or α-glucosylation, up to the linker unit that connects WTA with the PGN. Second, GlpQ is unable to cleave intact, unmodified LTA. Thus, WTA and LTA polymers of *B. subtilis* 168 constitute enantiomers consisting of Gro3P (WTA) and Gro1P (LTA) building blocks, respectively. Accordingly, limited hydrolysis of LTA with NaOH, which leads to random cleavage of phosphodiester bonds within the polymer, yields fragments that contain Gro3P terminal ends from which GlpQ is able to cleave off Gro3P entities. The difference in stereochemistry between WTA and LTA has critical consequences for the differential physiological functions, regulation, and turnover of both polymers. The results of this study rationalize the specific interaction of WTA and LTA with stereospecific enzymes and protection against simultaneous degradation with possibly fatal effects for cell viability.

## Experimental procedures

### Bacterial strains and growth conditions

The bacterial strains, plasmids, and oligonucleotides used in this study are listed in Table S1. The *B. subtilis* 168 WT and Δ*tagE::erm* strains were obtained from the Bacillus Genetic Stock Center (Columbus, OH). *B. subtilis* Δ*csbB::kan*, *L. monocytogenes* WT strain 10403S, and the Δ*gtlB::strep* mutant were obtained from the Gründling laboratory (38). These bacteria were used for isolation of whole cell wall (peptidoglycan–WTA complex) and teichoic acid preparations. They were cultured at 37 °C in lysogeny broth (LB; Lennox, Carl Roth) with continuous shaking at 140 rpm or on solid LB supplemented with 1.5% agar. Overnight cultures (∼16 h) were used to inoculate fresh LB medium and grown to yield an *A*_600_ of 1. Cells were harvested by centrifugation (3000 × *g*, 20 min, 4 °C). *E. coli* BL21 (DE3) cells (New England Biolabs) were used to heterologously express recombinant GlpQ phosphodiesterase from *B. subtilis*. These cells, transformed with pET28a-*glpQ*, were grown in LB medium supplemented with 50 μg/ml kanamycin until *A*_600_ 0.7 was reached, followed by induction with 1 mm isopropyl 1-thio-β-d-galactopyranoside and further propagation for 3 h. Cells were harvested by centrifugation (3000 × *g*, 20 min, 4 °C) and used for purification of recombinant GlpQ.

### Construction of plasmids and purification of recombinant GlpQ

*B. subtilis* 168 *glpQ* was amplified by PCR with the primers pET28a-glpQ-for and pET28a-glpQ-rv (MWG Eurofins, Ebersberg, Germany). Oligonucleotide primers are listed in Table S1. The PCR products were purified (Gene JET Purification Kit and Gene Ruler, 1-kb marker, Thermo Fisher Scientific), digested with the appropriate restriction enzymes (New England Biolabs), and ligated with T4 DNA ligase (Thermo Fisher Scientific) into the expression vector pET28a (Novagen), allowing them to overproduce a C-terminal His_6_ tag fusion protein. *E. coli* BL21 (DE3) cells carrying pET28a-*glpQ* were grown as described above and lysed in a French pressure cell. The His-tagged GlpQ protein was purified by Ni^2+^ affinity chromatography using a 1-ml HisTrap column (GE Healthcare), followed by size exclusion chromatography on a HiLoad 16/60 Superdex 200 pg column (GE Healthcare), and purity was checked with 12% SDS-PAGE. The purity of the enzyme was confirmed via SDS-PAGE (Fig. 2*A*). From a 1-liter culture, 3.6 mg of GlpQ was obtained. The enzyme was stored at a concentration of 0.23 mg/ml at −20 °C in 0.1 m Tris-HCl buffer (pH 8).

### Biochemical characterization of GlpQ

To determine the enzymatic properties of GlpQ, 1 pmol of pure recombinant enzyme was incubated with 10 mm GPC. The reaction was stopped by adding 200 μl of pH 3.3 buffer (0.1% formic acid and 0.05% ammonium formate), and the released glycerolphosphate was measured by HPLC-MS. For pH stability, GlpQ was preincubated in buffers at different pH values (pH 2, HCl; pH 3–6, acetic acid; pH 6–7, MES; pH 7–9, Tris; pH 10, NaHCO_3_) for 30 min at 30 °C before adding 5 μl of each to a 45-μl mixture with 0.1 m Tris (pH 8) buffer and substrate for 5 min. The pH optimum was tested by incubating GlpQ with 10 mm GPC for 5 min in buffers with different pH values. For temperature stability, GlpQ was preincubated in 0.1 m Tris-HCl (pH 8) at different temperatures ranging from 4 °C to 75 °C for 30 min, followed by 5-min incubation with GPC at 30 °C and pH 8.0. The optimum temperature was tested by incubating GlpQ for 5 min at different temperatures with GPC at pH 8.

### Preparation of cell walls, WTA, and LTA

For the preparation of cell walls (peptidoglycan–WTA complex), 2 liters of *B. subtilis* 168 WT or Δ*tagE::erm* cultures (exponential growth phase, *A*_600_ = 0.9) were harvested and resuspended in 30 ml of piperazine acetate buffer (50 mm (pH 6)) with 12 units of proteinase K and boiled for 1 h. The cytosolic fractions were removed by centrifugation (3000 × *g*, 15 min, 4 °C). The pellet was resuspended in 6 ml of buffer (10 mm Tris, 10 mm NaCl, and 320 mm imidazole, adjusted to pH 7.0 with HCl), and 600 μg of α-amylase, 250 units of RNase A, 120 units of DNase I, and 50 mm of MgSO_4_ were added. The sample was incubated at 37 °C for 2 h while shaking, and then 12 units of proteinase K was added, and the incubation continued for 1 h. 4% SDS solution was added 1:1, and the mixture was boiled for 1 h. SDS was removed by repeated ultracentrifugation steps (20 times at 140,000 × *g*, 30 min, 40 °C), suspension in double-distilled H_2_O, as well as dialysis against double-distilled H_2_O. The SDS content was controlled with a methylene blue assay described earlier (54). The cell wall preparation was dried in a vacuum concentrator. LTA from *B. subtilis* 168 (WT and Δ*csbB*) and *L. monocytogenes 10403S* (WT and Δ*gtlB*) was prepared by butanol extraction and purification by hydrophobic interaction chromatography using a 24 × 1.6-cm octyl–Sepharose column, according to published protocols (51, 52).

### Teichoic acid digestion with GlpQ and analysis of glycerolphosphate release

WTA assays were conducted in 0.1 m Tris-HCl buffer (pH 8, supplemented with 1 mm CaCl_2_) with 0.1 mg cell wall preparation (peptidoglycan with attached WTA from *B. subtilis* 168 WT and Δ*tagE::erm*) as a substrate and 0.7 μm GlpQ. The samples were incubated for 30 min at 30 °C.

LTA assays occurred in 0.1 m Tris-HCl buffer (pH 8, supplemented with 1 mm CaCl_2_) with 0.2 mg LTA extract (*B. subtilis* 168 WT and Δ*csbB::erm*) and 0.7 μm GlpQ in a total volume of 50 μl. The samples were incubated for 1 h at 30 °C. LTA was predigested by incubation with 0.1 m NaOH for 30 min at 60 °C, followed by neutralization with HCl and drying in a vacuum concentrator.

Sample analysis was conducted using an electrospray ionization–TOF mass spectrometer (MicrOTOF II, Bruker Daltonics) operated in positive ion mode and connected to an UltiMate 3000 HPLC system (Dionex). For HPLC-MS analysis, 7 μl of the sample supernatant was injected into a Gemini C18 column (150 by 4.6 mm, 5 μm, 110 Å, Phenomenex). A 45-min program at a flow rate of 0.2 ml/min was used to separate the compounds as described previously (55). The mass spectra of the investigated samples were presented as base peak chromatograms (BPCs) and extracted ion chromatograms (EIC) in the DataAnalysis program and presented by generating diagrams using Python 3.6 with the Matplotlib (version 2.2.2) library.

## Author contributions

A. W. and C. M. conceptualization; A. W., J. R., and A. G. resources; A. W. data curation; A. W. and S. U. formal analysis; A. W. validation; A. W. and S. U. investigation; A. W. visualization; A. W., S. U., J. R., and A. M. J. methodology; A. W. writing-original draft; A. P., A. G., and C. M. supervision; A. P., A. G., and C. M. funding acquisition; A. P., A. G., and C. M. writing-review and editing; C. M. project administration.

## Supplementary Material

Supporting Information

## Footnotes

5 The abbreviations used are:

PGN: peptidoglycan
ManNAc: *N*-acetylmannosamine
PG: phosphatidylglycerol
DAG: diacylglycerol
PGP: phosphatidylglycerol phosphate
Gro3P: *sn*-glycerol-3-phosphate
Gro1P: *sn*-glycerol-1-phosphate
GPC: *sn*-glycero-3-phosphocholine
AUC: area under the curve
LB: lysogeny broth
BPC: base peak chromatogram
EIC: extracted ion chromatogram.
