# Supplementary material for: Phosphoglycerol-type wall and lipoteichoic acids are enantiomeric polymers differentiated by the stereospecific glycerophosphodiesterase GlpQ
Source: J Biol Chem. 2020 Feb 11;295(12):4024–34. doi: 10.1074/jbc.RA120.012566 (PMC7086022; doi:10.1074/jbc.RA120.012566)
Supplement: Supporting Information [file supp_295_12_4024__index.html]

Phosphoglycerol-type wall and lipoteichoic acids are enantiomeric polymers differentiated by the stereospecific glycerophosphodiesterase GlpQ — Stereochemistry of Teichoic Acids — Supporting Information 

# Phosphoglycerol-type wall and lipoteichoic acids are enantiomeric polymers differentiated by the stereospecific glycerophosphodiesterase GlpQ

## Supporting Information

- Supporting Information (to be published online) - Supporting figures and table
