# Supplementary material for: Phosphoglycerol-type wall and lipoteichoic acids are enantiomeric polymers differentiated by the stereospecific glycerophosphodiesterase GlpQ
Source: J Biol Chem. 2020 Feb 11;295(12):4024–34. doi: 10.1074/jbc.RA120.012566 (PMC7086022; doi:10.1074/jbc.RA120.012566)
Supplement: Supporting Information [file supp_RA120.012566_157866_2_supp_473444_q5j6tm.docx]

**Supporting Information to**

Phosphoglycerol-type wall- and lipoteichoic acids are enantiomeric polymers
differentiated by the stereospecific glycerophosphodiesterase GlpQ

**Axel Walter^#^, Sandra Unsleber^#^, Jeanine Rismondo^§^, Ana Maria Jorge^¶^, Andreas Peschel^¶^,
Angelika Gründling^§^, Christoph Mayer^#^**

From ^#^Microbiology/Glycobiology and **^¶^**Infection Biology, Interfaculty Institute of Microbiology and Infection Medicine Tübingen (IMIT), University of Tübingen, 72076 Tübingen, Germany and the ^§^Section of Molecular Microbiology and Medical Research Council Centre for Molecular Bacteriology and Infection, Imperial College London, London SW7 2AZ, United Kingdom

**Supporting Figures**


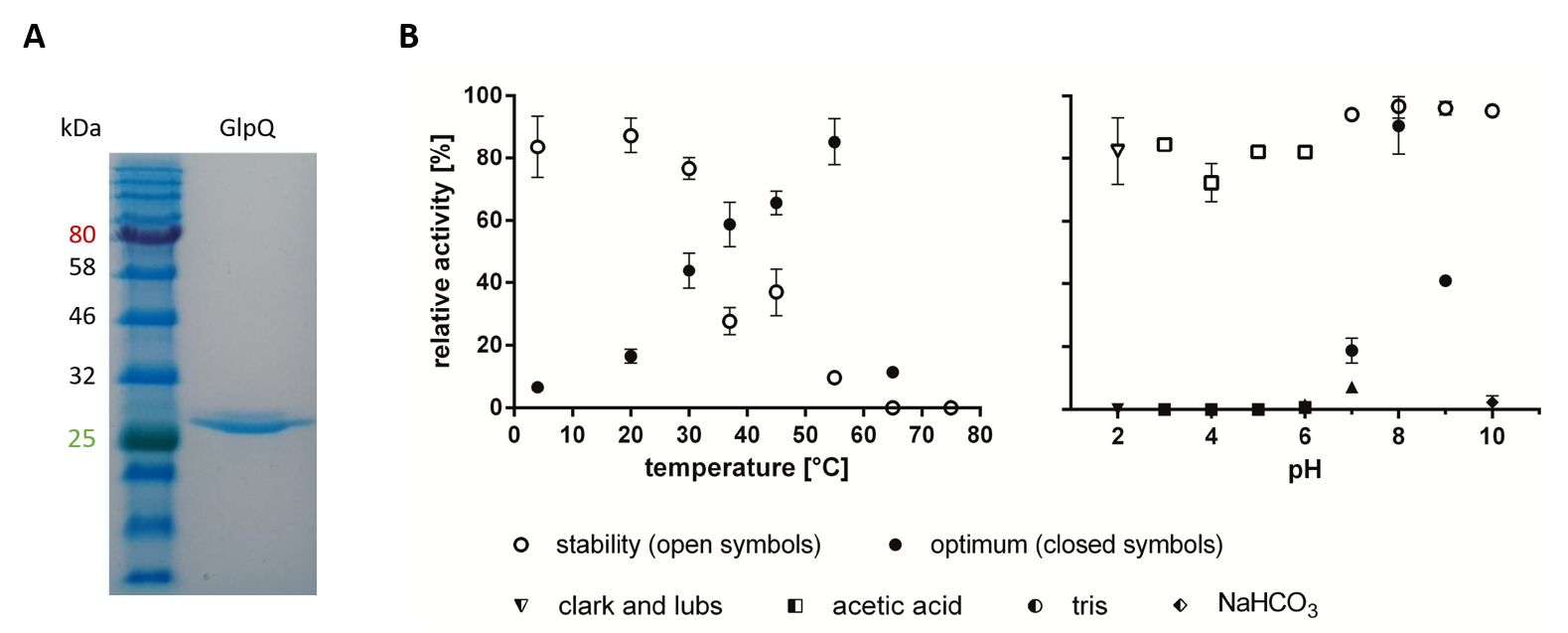


**Figure S1.  Enzyme purity, stability and optima of recombinant GlpQ.** *A*, SDS-PAGE of heterologously expressed recombinant GlpQ-His_6_ fusion protein (GlpQ) after purification of the enzyme by Ni^2+^ affinity chromatography and size exclusion chromatography (1 μg protein was loaded on a 12% polyacrylamide gel). A protein band is visible that migrates similar to the 25 kDa marker protein, in agreement with the calculated molecular weight of GlpQ of 29.6 kDa. *B*, Temperature and pH characteristics of GlpQ. The enzyme is stable for 30 min at temperatures up to 30°C, but stability rapidly decreases at temperatures above 30°C within this time frame. Activity of GlpQ increases with temperature up to 55°C with half maximum activity at 30°C. GlpQ is stabile within a broad range between pH 2-10 in the indicated buffers and has a very sharp pH-optimum at 8.0. In all assays, 1 pmol GlpQ was incubated with 10 mM GPC and the reaction product was analysed by LC-MS, after 30 min of incubation at 30°C. For temperature stability and optimum, 100% relative activity reflect area under the curve (AUC)-values of 2240 and 4088, respectively. For pH stability and pH optimum 100% AUC were 28571 and 31903, respectively.

*
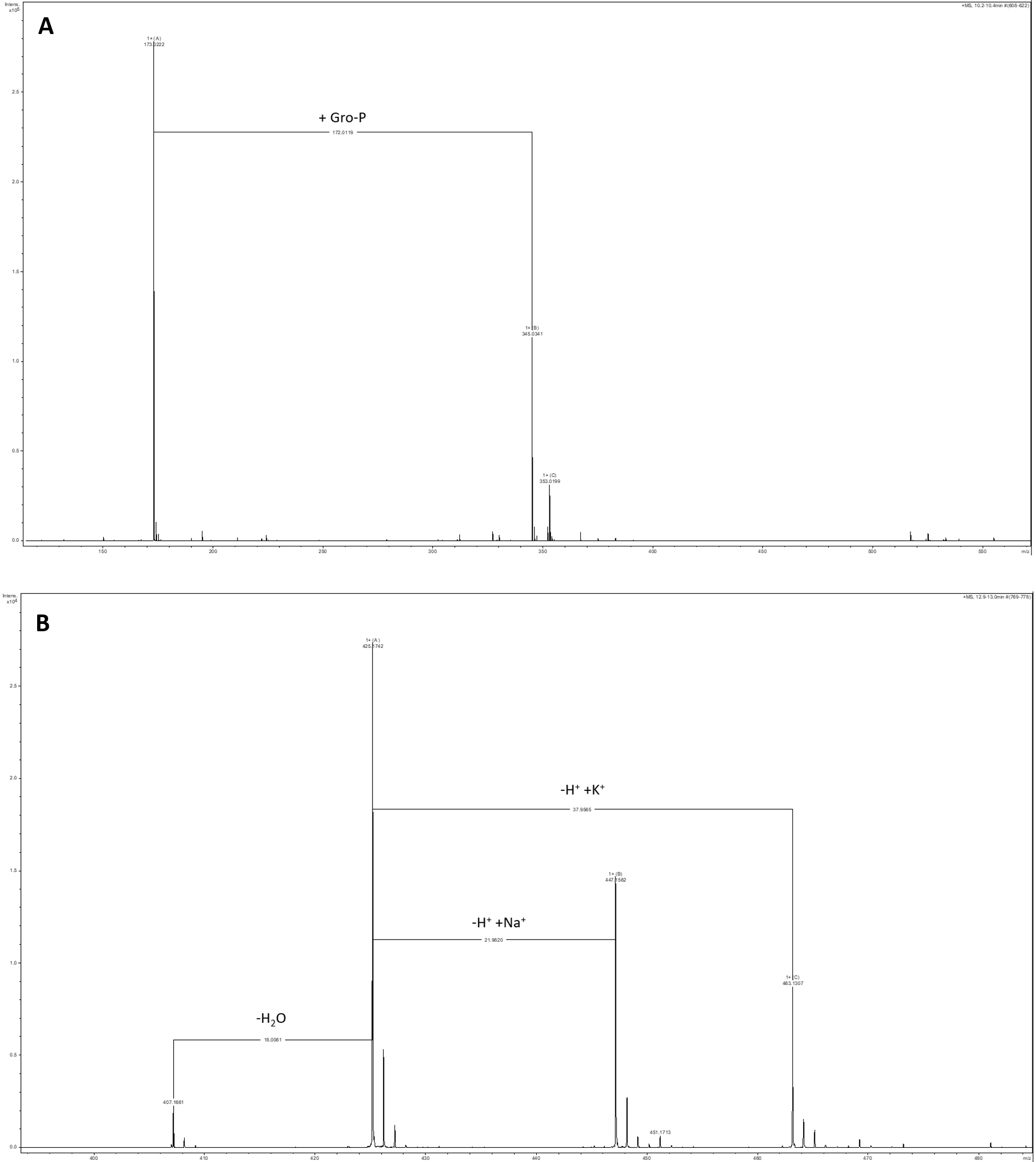
*

**Figure S2.  Mass spectra of glycerolphosphate (GroP) and the WTA linker disaccharide ManNAc-β-1,4-GlcNAc.** *A,* Mass spectrum of GroP analysed in positive ion mode [M+H]^+^ (experimental 173.0222; theoretical monoisotopic mass 173.0210), also revealing a non-covalently bound GroP dimer, [2M+H]^+^ (experimental 345.0341; theoretical 345.0346). *B*, Mass spectrum of the WTA linker disaccharide, ManNAc-GlcNAc, analysed in positive ion mode [M+H]^+^ (experimental 425.1742; theoretical monoisotopic mass 425.1766), also revealing the sodium adduct [M+Na]^+^ (experimental 447.1562; theoretical 447.1585), the potassium adduct [M+K]^+^ (experimental 345.0341; theoretical 345.0346), as well as a product of neutral water loss, [M-(H_2_0)+H]^+^ (experimental 407.1661; theoretical 407.1660) .


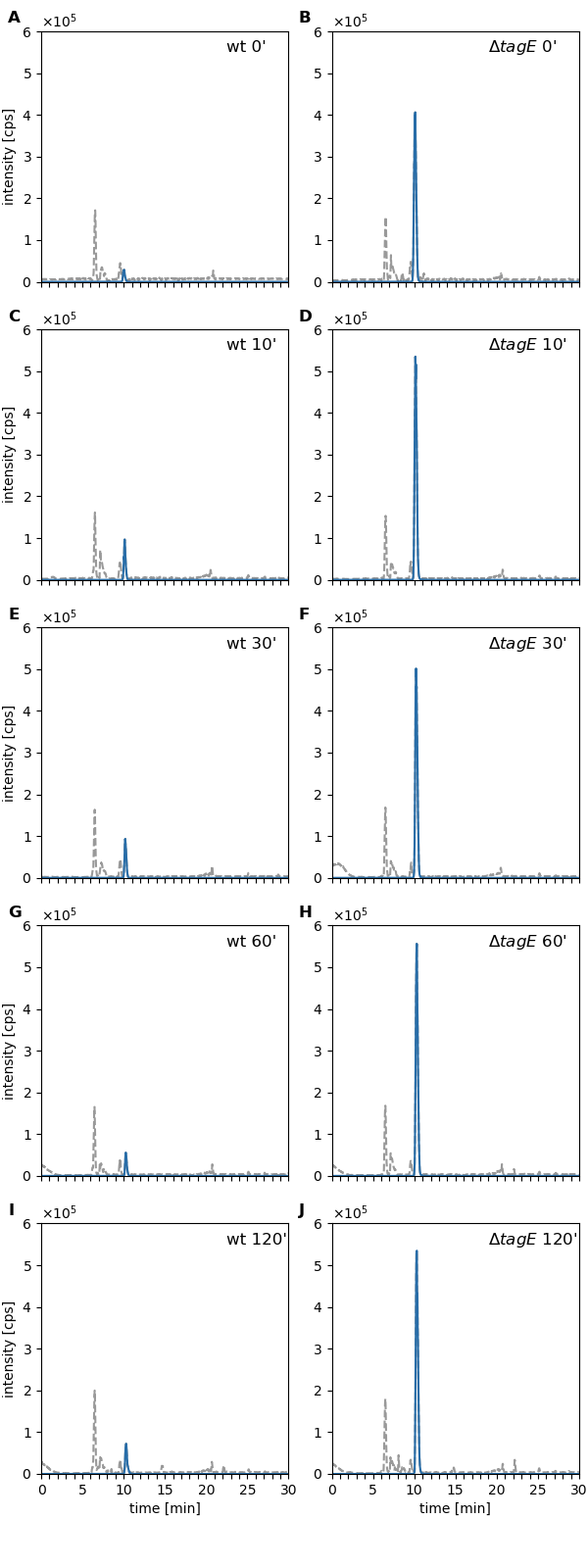


**Figure S3. Time course of WTA digestion by GlpQ.**  The vast amount of product (GroP; blue lines) is released by GlpQ within the first seconds of incubation of cell walls purified from wild-type cells (wt; *A,C,E,G,I;* incubation time in min) and non-glycosylated cell wall (from *ΔtagE* cells; *B,D,F,H,J;* incubation time in min). GlpQ releases significantly more product from non-glycosylated (from *ΔtagE* cells) than from the glycosylated wild-type substrate. Even over a long period of time no more GroP is released from wild-type cell wall, indicating that GlpQ has only exo- and no endo-lytic activity. 0.25 mg purified cell wall of *B. subtilis* (containing PGN and covalently bound WTA) was incubated with 0.7 µmol GlpQ and the formation of reaction products was analysed by LC-MS. Shown are the base peak chromatograms (BPC) mass range (M+H)^+^ = 120 – 800 (gray dashed) and the extracted ion chromatograms (EIC) of GroP (M+H)^+^ m/z = 173.022 +/- 0.02 (blue solid). The reaction was stopped by incubation at 95°C followed by LC-MS analysis.

**
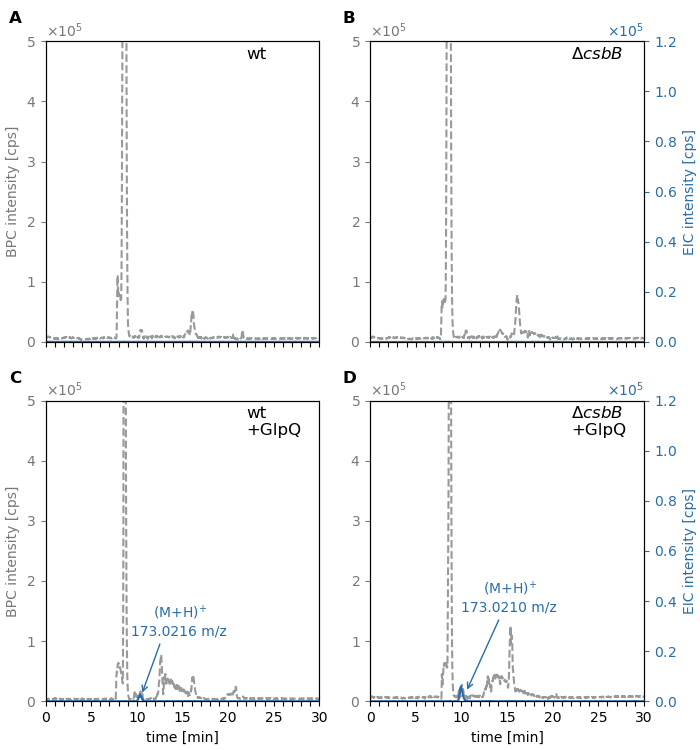
**

**Figure S4. *B. subtilis* 168 LTA not preincubated at pH 8 for 24 h cannot be cleaved by GlpQ.** Purified *B. subtilis* LTA was incubated with GlpQ and the formation of reaction products was analysed by LC-MS. Very little amounts of GroP were released by GlpQ. ***A*** and ***C***, wild-type (wt) LTA (= partially glycosylated LTA) incubated without GlpQ (control) with GlpQ. The peak area of released GroP was AUC = 6 x 10^4^. *B* and *D*, non-glycosylated Δ*csbB* LTA incubated without GlpQ (control) and with GlpQ. The peak area of relased GroP was AUC = 1.4 x 10^5^. Shown are the base peak chromatograms (BPC) mass range (M+H)^+^ = 120 – 800 (gray dashed) and the extracted ion chromatograms (EIC) of glycerol-phosphate (M+H)^+^ m/z = 173.022 +/- 0.02 (blue solid).

**
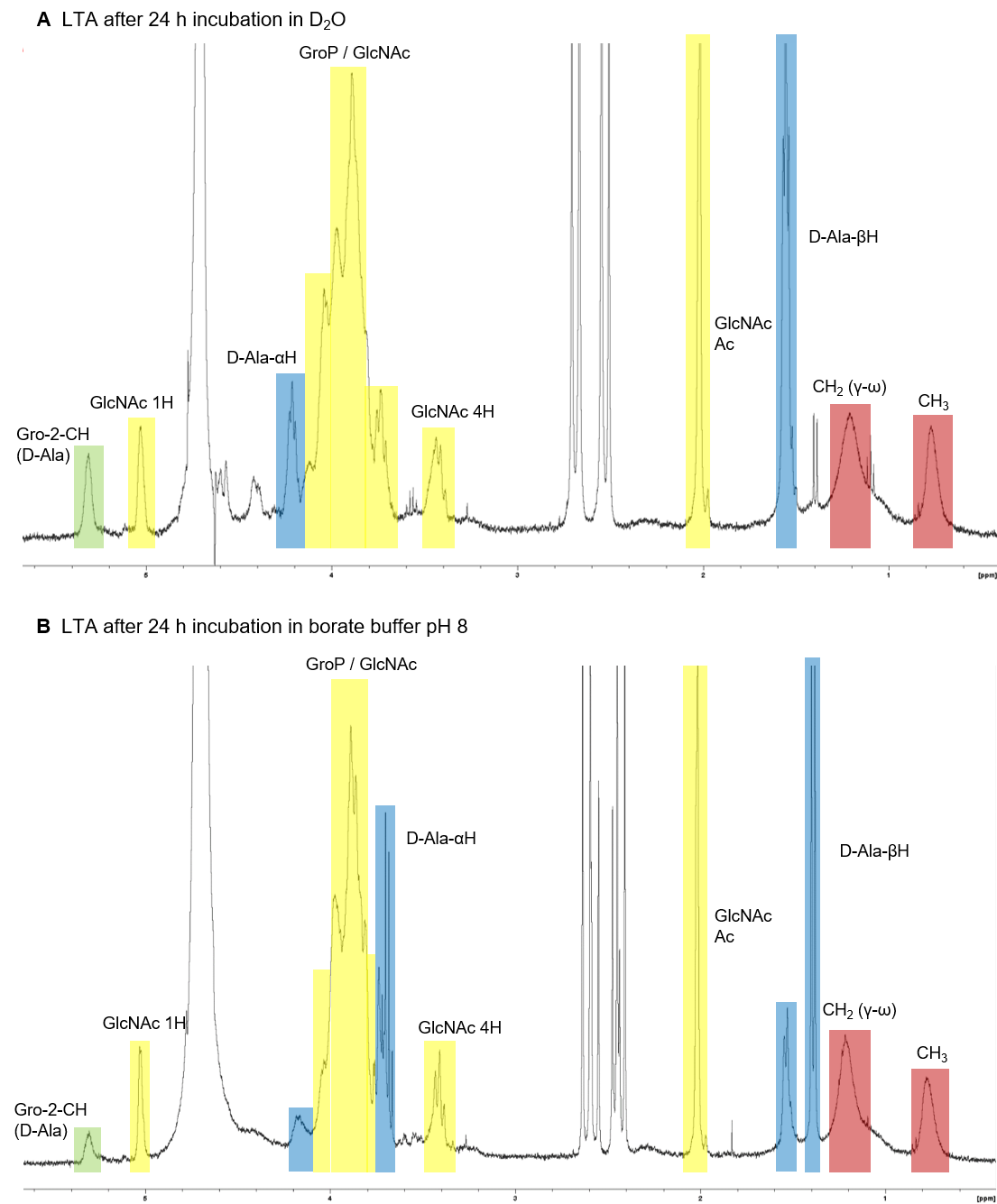
**

**Figure S5. ^1^H-NMR analysis of LTA following incubation at pH 8 for 24 h.** Shown are the **^1^**H-NMR spectra (400 MHz, 303K) of LTA isolated from *B. subtilis* 168 wt (2 mg) either incubated for 24 h at room temperature in *A*, distilled water (D_2_O), pH 7.0, or in *B*, D_2_O containing 0.1 M borate buffer pH 8.0. Color coding identifies signals indicating removal of D-alanyl residue from the GroP polymer (green): the resonance of the methine group of *sn*-glycerol (Gro-2-CH) containing an D-alanyl ester (D-Ala) is reduced and partially shifted from 5.3 ppm to 3.9 ppm and the D-Ala-ɑH and D-Ala-βH resonances (blue) are significantly reduced and partially shifted in the LTA sample incubated at pH 8 compared to LTA in D_2_O. Other resonances assigned to GlcNAc substitution and the GroP polymer (yellow) and to the fatty acids of LTA (red) are not influenced by incubation in borate buffer at pH 8.0. From the signal integral estimation, about two-thirds of the D-Ala substituents are removed in the LTA sample by incubation at pH 8 for 24 h. NMR analysis was performed on a 400-MHz Bruker Advance III spectrometer at 303 K with a TCl cryoprobe. NMR spectra were interpreted according to (1).

**Supporting Table**

**Table S1. Strains, plasmids and primer used in the study**

| **Strain or plasmid** | **Characteristics** | **References** |
| --- | --- | --- |
| ***E. coli*** |  |  |
| BL21 (DE3) | *fhuA2 [lon] ompT gal (λ DE3) [dcm] ΔhsdS λDE3 = λ sBamHIo ΔEcoRI-B int::(lacI::PlacUV5::T7 gene1)*  *i21 Δnin5* | New England Biolabs |
| ***B. subtilis*** |  |  |
| strain 168 (wild-type) | *trpC2*; genome sequenced *B. subtilis* type strain | *Bacillus* Genetic  Stock Center |
| Δ*tagE::erm* | 168; *trpC2, tagE* exchanged by erm^R^  with flanking *loxP* sites | *Bacillus* Genetic  Stock Center |
| Δ*csbB::kan* | 168; *ΔcsbB::kan* | (2) |
| ***L. monocytogenes*** |  |  |
| strain 10403S  (wild-type) | 10403S; StrepR | (3) |
| *∆gtlB::strep* | 10403S; *∆gtlB*; StrepR | (2) |
| **plasmids** |  |  |
| pET28a | KanR, T7 promoter, ori pBR322, lacI | Novagen |
| pET28a-*glpQ* | KanR, T7 promoter, ori, pBR322,lacI,  adds C-terminal His_6_-tag to *glpQ* | this work |
| **primer** |  |  |
| pET28a-glpQ-for | GATATACCATGGTGGCGTCAAAAGGAAACCTGC | this work |
| pET28a-glpQ-rv | GTGGTGCTCGAGATAACCCTTTTTTACTTTGTGGA | this work |

**References**

1. Morath, S., Geyer, A., and Hartung, T. (2001) Structure-function relationship of cytokine induction by lipoteichoic acid from *Staphylococcus aureus*. *J Exp Med* **193**, 393-397

2. Rismondo, J., Percy, M. G., and Gründling, A. (2018) Discovery of genes required for lipoteichoic acid glycosylation predicts two distinct mechanisms for wall teichoic acid glycosylation. *J Biol Chem* **293**, 3293-3306

3. Webb, A. J., Karatsa-Dodgson, M., and Gründling, A. (2009) Two-enzyme systems for glycolipid and polyglycerolphosphate lipoteichoic acid synthesis in *Listeria monocytogenes*. *Mol Microbiol* **74**, 299-314
